# ‘Picking up the pieces’: primary care practitioners’ experiences of cancer care reviews. A descriptive qualitative study

**DOI:** 10.3399/BJGPO.2024.0064

**Published:** 2025-01-15

**Authors:** Dipesh P Gopal, Stephanie JC Taylor, Ping Guo, Nikolaos Efstathiou

**Affiliations:** 1 Centre for Primary Care, Wolfson Institute of Population Health, Barts and the London School of Medicine and Dentistry, Queen Mary University of London, London, UK; 2 School of Nursing and Midwifery, Institute of Clinical Sciences, University of Birmingham, Birmingham, UK

**Keywords:** living with and beyond cancer, cancer care reviews, general practice, cancer, primary healthcare

## Abstract

**Background:**

The number of people who are living with and beyond cancer is increasing in England. Primary care delivers cancer care via structured proactive conversations which are incentivised through the Quality and Outcomes Framework (QoF): ‘cancer care reviews’ (CCRs). Declining workforce numbers, increasing patient demand, CCR policy changes in 2020, and the onset of the coronavirus disease 2019 (COVID-19) pandemic motivate exploration of how staff deliver CCRs.

**Aim:**

To explore primary care staff’s experiences with CCRs, their view of CCRs, how they conduct CCRs, and their perception of the value of CCRs.

**Design & setting:**

Descriptive qualitative study in general practices in England.

**Method:**

Semi-structured online interviews with 15 primary care staff; data analysis using reflexive thematic analysis.

**Results:**

Four themes were identified: varied and evolving perception of cancer; the delivery and impact of CCRs; changes to CCR delivery during the COVID-19 pandemic; and ways to complement CCRs. Primary care staff felt that the way that cancer was perceived by patients, including those from ethnic minority backgrounds, impacted how CCRs were delivered. Cancer care involved acknowledging the challenge of a cancer diagnosis, helping decode jargon, and addressing unmet care needs. The pandemic resulted in remote CCR delivery for some practices. Staff suggested that community cancer teams could provide cancer care alongside existing services.

**Conclusion:**

Staff adopted the new 3- and 12-month format CCRs despite the COVID-19 pandemic. Clinical staff may benefit from better training on cancer as a long-term condition and how cancer is perceived by people from diverse ethnic backgrounds.

## How this fits in

There is limited evidence to show the benefit of financial incentivised conversations between English primary care clinicians and patients with a recent diagnosis of cancer, known as cancer care reviews (CCRs). This study explored the experiences of 15 clinicians delivering CCRs. Clinicians provided holistic care by navigating different duties: signposting to relevant services; decoding clinical letters; and filling gaps in care. Financial incentives helped achieve care standards, but cancer care was delivered regardless of when clinical need arose throughout the year. Community cancer care teams were suggested as a solution to help provide better integrated care across different sectors.

## Introduction

The number of people who are living with and beyond cancer is increasing in the UK and worldwide. Globally, by 2040 there will be over 28 million people with a cancer diagnosis, which is a 47% rise from 2020.^1^ ‘Living with and beyond cancer’ is a term including people who have a cancer diagnosis and may be having, or have completed, cancer treatment.^2^ In the UK, 3 million people were living with and beyond cancer in 2020, which will increase to 4 and 5.3 million in 2030 and 2040 respectively.^3^ A 50% increase in cancer survival in England and Wales from 1970 to 2014 is probably the result of improved diagnosis and treatment.^4^ Increased survival is associated with challenges, which may be physical, such as tiredness; psychological, such as depression; sexual, such as loss of libido; and social, such as poverty and loss of income (termed ‘financial toxicity’^5^) or the impact on relationships.^6^

Primary care staff in England support people with recent diagnoses of cancer via structured proactive conversations which are incentivised through the Quality and Outcomes Framework (QoF). These are called ‘cancer care reviews’ (CCRs). This provides an opportunity for patients to discuss their concerns, understand relevant community support, and be supported in healthy behaviour change.^7^ CCRs occur in the context of wider support initiated by secondary care services and occur in two parts: a 3-month and 12-month review.^8^ A 3-month CCR provides information about support, whereas the 12-month CCR is a consultation.^9^ Some primary care staff continue annual CCRs despite there being no financial incentive to do so after the first year. However, there is insufficient evidence to demonstrate the impact of CCRs on patient symptoms, quality of life, and outcomes.^10^ Since 2015, there has been limited research examining CCR delivery in primary care. The last surveys of primary care staff’s views on CCRs were published in 2010 and 2015, and the last focus group in 2011.^11–13^ Since 2015 there has been a 4.4% decrease in full-time equivalent (FTE) GPs and since 2014, there has been an 8.3% increase in FTE practice nurses, while the numbers of patients per practice have increased by 24%.^14–17^ Working hours have increased, with the primary care workforce managing existing workloads alongside the COVID-19 vaccination drive since the pandemic.^17^ There is a need for up-to-date research, given the workforce changes, to appreciate primary care professionals’ views on delivering CCRs. This aligns with the top James Lind Alliance research priority: identifying the best models for long-term cancer care.^18^ To understand the impact and context of CCRs in primary care, patient and public representatives were contacted via a regional participant group for people with a cancer diagnosis and a local cancer charity before study set-up. Representatives noted the absence of primary care in their cancer care and a feeling of abandonment after discharge from secondary care.

The aim of this study was to explore general practice staff’s views of CCRs, their experiences of conducting CCRs, as well as the perceived impact on and value of CCRs for patients and their families.

## Method

### Design

A qualitative study design was undertaken using a descriptive approach^19^ within a critical realist ontology and interpretivistic epistemology. This study was reported using the Standards for Reporting Qualitative Research (SRQR).^20^

### Recruitment

General practice staff who conducted CCRs were recruited via professional network WhatsApp groups, and social media posts on LinkedIn and Instagram. A mixture of purposive and snowball sampling ensured a diversity in professional backgrounds, clinical experiences, seniority, sex, and ethnicity. Thirty-two primary care staff were approached via email with a participation information sheet and consent form after initially expressing interest via WhatsApp or social media.

A sample size of 15 participants promoted data adequacy,^21^ to include both confirming and disconfirming data, and was informed by a systematic review suggesting that 12–13 interviews are usually required to reach code and theme saturation.^22^

### Data collection and storage

A participation information sheet was provided and written informed consent was gained before interview. Semi-structured online interviews were conducted via Zoom video conferencing software during June and July 2022. A topic guide was developed and initially piloted with three interviews, which were included in the final analysis, but it did not change iteratively throughout the study (Supplementary Information S1). Interviews were conducted by the first author (DPG) and audio-recorded, and automatically transcribed by Zoom software. The auto-transcribed interviews were checked for accuracy by DPG who maintained a study diary to promote reflexivity. The first three interviews were discussed, and jointly analysed with co-authors for richer interpretation of meaning in data^23^ before proceeding to the remaining interviews. The data were stored on secure servers, and audio files were deleted after the individual interview transcripts were checked for accuracy. The transcripts were anonymised.

### Data analysis

Reflexive thematic analysis was used to derive inductive themes after data collection^24,25^ using six steps:

1. Familiarisation with the data
2. Generating initial codes
3. Searching for themes
4. Reviewing themes at the level of coded extract and data set to create maps
5. Defining, naming, and revising themes
6. Ensuring thematic analysis answers research questions

NVivo (version 12) was used initially to derive codes from the interview transcripts, line-by-line, before presentation and discussion of the initial themes to co-authors who had experience in reflexive thematic analysis. A mixture of primary care and secondary care clinical experience as a GP as well as intensive care and oncology nursing provided rich discussion before agreement on themes and subthemes, to ensure thematic analysis addressed the research aim.

## Results

Fifteen primary care staff familiar with CCRs were interviewed and participant characteristics are summarised in Table 1. Staff worked clinically for 2.5±1 working days (mean±standard deviation) and spent the remainder of the week in other roles. Clinical experience in general practice varied from 6 months to 30 years, with mean experience of 11±9 years.

**Table 1.** Interviewee demographic characteristics

Demographic characteristic	Frequency, *n* (%)
**Mean age, years±SD**	42±10
**Sex**	
Female	12 (80%)
Male	3 (20%)
**Ethnicity**	
White	9 (60%)
British South Asian (Indian, Pakistani, Nepali)	5 (33%)
Black African	1 (7%)
**Occupation**	
GP	11 (73%)
Practice nurse	3 (20%)
Physician associate	1 (7%)
**Other non-clinical roles**	
Managerial – practice specialty lead	5 (33%)
Managerial – practice, managing partner	5 (33%)
Managerial – clinical commissioning group (CCG) / regional	5 (33%)
Teaching – undergraduate	3 (20%)
Teaching - postgraduate	5 (33%)
Research	2 (13%)

SD = standard deviation.

The self-reported mean population size of the GP practices in which participants worked was 17600. Most participants reported working in urban settings (*n*=10) while others were in suburban (*n* = 4) and rural settings (*n* = 1). Most practices served mixed affluent and deprived communities (*n* = 9) and ethnically diverse populations (*n* = 10).

**Figure 1.**
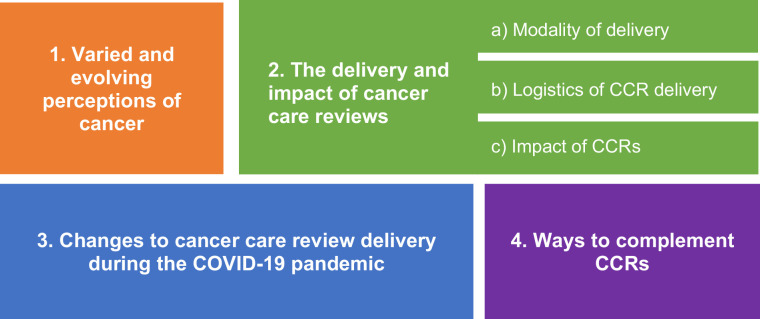
Summary of themes and subthemes. CCR = cancer care review. COVID-19 = COronaVIrus Disease 2019

Four themes were developed (see Figure 1): varied and evolving perceptions of cancer; delivery and impact of CCRs; changes to delivery of CCRs during the COVID-19 pandemic; and ways to complement CCRs.

### Theme 1: varied and evolving perceptions of cancer

The ways that cancer was viewed by different participants varied markedly. Some primary care staff viewed cancer like a long-term condition and conducted CCRs in chronic disease clinics:

'... *we're trying to embed a system where* [we] *treat it like a chronic disease, like all the other chronic diseases that practice nurses deal with* ... ' (Practice nurse, 010)

It was evident that increasing complexity in different cancer types and treatments made it difficult for some primary care staff to provide holistic cancer care via CCRs:

*'I think it’s also difficult because ... cancer is such a big umbrella term ... and there’s such a diverse range of diseases ... And … oncologists also aren’t specialists in every area of cancer, right ... but even they specialise in one area of cancer*.' (Locum GP, 001)

Furthermore, some clinicians noted how cancer may be viewed differently by people with diverse cultural, religious, and spiritual backgrounds, and that this might impact the way CCRs are delivered:

' ... *we have to now start thinking of cultural humility to say that ‘one size does not fit all of us’, and we need to interpret it based on that person’s health belief, culture, religion ... Some people believe in miracles as well ... that they are cured of* [cancer]*. So, you then coming in and saying you know "your cancer ... you've got to treat this and treat that" ... it’s how to navigate it and support them around their beliefs* ... ' (GP Partner, 005)

### Theme 2: the delivery and impact of cancer care reviews

#### Modality of delivery

How CCRs were conducted depended on patient convenience and what was offered by primary healthcare staff:

*' ... but a lot of the 3-month ones tend to be over the phone. Because they've got a lot of hospital appointments ... they don't want another one … I’d offer them both but … the 12-month ones tend to be face-to-face.*' (Practice nurse, 009)

While some consultations occurred remotely, primary care staff noted the importance of in-person consultations as they help with communication, facilitating empathy and the picking up of visual clues:

' ... *you could see how they interact with a relative, or ... how they walk into your consulting room, or ... whether they're still smoking ... little things you can pick up that are actually quite important to their care needs, and prognosis* ... ' (Locum GP, 001)

#### Logistics of CCR delivery

In preparation for the CCR appointment, primary care staff sent out questionnaires, concerns checklists, or holistic needs assessments via a text messaging platform, ‘Accurx’, to help identify patient concerns. Some staff tailored clinical care before appointments by reading through consultation notes and clinical letters while considering the social context of the patient:

*'And I think it might take five to ten minutes to even figure out how long do I need to spend with this patient? Or maybe even do a little bit of … preparation for such a review. And it’s definitely important for me to ask these X, Y, Z questions to the patient*.' (Salaried GP, 015)

During the appointments for CCRs, primary care staff navigated several duties such as treating physical symptoms and mental health problems, and performing monitoring blood tests such as prostate-specific antigen (PSA). Staff also completed non-CCR activities such as administering hormone treatments, providing lifestyle advice, and advising on signs of cancer recurrence, as well as discussing sexuality after cancer treatment:

*'We talked about sexuality, whether or not he’s had an erection yet, because one of the nerves was left intact. How that was affecting him and his wife and their personal relationship and their sexual relationship.*' (Practice nurse, 009)

Some participants reported decoding jargon within oncology letters and helping patients understand treatment options during CCRs:

*'Most letters that you get from the oncology team, for instance, there will be like six or seven lines and it’s just numbers and letters. Sometimes even, I have to ‘Google’ it ... And for a patient is probably just really, really scary and impenetrable to know what’s going on*.' (Salaried GP, 015)

Primary care staff saw their role as a care coordinator between primary, secondary, and palliative care services. This included signposting to online support, charities and local services, such as Citizens Advice Bureau and local support groups:

*'Say if someone’s working and they're worried about finances, we have a social prescriber here at surgery … she can chat to them about the benefits support and advice. If we catch someone that’s maybe living on their own, and we think might struggle, then I would refer them social services*.' (Practice nurse, 004)

Filling informal *‘gaps’* in support was termed *‘picking up the pieces’*; examples of this were an unmet care need or ‘lost’ referrals:

*'If we’re doing our job, we’re the ones picking it up early, we’re the ones getting them on a two week wait, we’re the ones that have picked it up, we’re the ones are going to pick up the pieces when they’ve got the diagnosis and then just check in while they’re having their formal treatment*.' (GP Partner, 012)

In general, CCRs were a small part of wider cancer care delivered annually. While CCRs are financially incentivised during in the first year of treatment, staff vocation ensured cancer care was delivered beyond those financial incentives:

' ... *so if you're following QoF, it’s once a year. But like I said, if you’ve got a patient that is becoming palliative and needs a few more other things done. You’re not going stick to once a year, are you?*' (GP Partner, 005)

Templates guaranteed a care standard but were limited by their inflexibility:

*'Because you want to ... make sure you cover everything … Of course, you don’t want important things to be missed. So, I guess what’s good about template but then it doesn’t allow individual exploration of things that are more important to that person at that particular time*.' (Locum GP, 001)

#### Impact of CCRs

Primary care staff thought some patients benefited from CCRs and were grateful for a proactive appointment which provided structure after a cancer diagnosis:

*'You know it feels like there’s things that they’re going to discuss, and they have an opportunity to bring things up, and I think having that structure can sometimes be helpful in a cancer world ... well, that can seem a little bit out of control*.' (Locum GP, 001)

CCRs provided acknowledgement of the challenge of a cancer diagnosis or treatment:

*'I think it’s … just acknowledging that you’re aware it’s happening, and you know come to us if we* [sic] *need anything, that type of conversation. The patients are very happy to receive the call*.' (Practice nurse, 004)

Despite this, some patients declined the offer of a CCR because they did not want to adopt the ‘sick role’ or allow a cancer diagnosis to dictate their life course:

*'I think you can get a little bit annoyed even by this whole process because they don’t want the cancer, to take over their life or to kind of be seen to be a cancer patient. This is a part of my life and I’m just carrying on with it*.' (Salaried GP, 015)

It was difficult to ascertain the impact of CCRs on family members or friends as this is not a formal part of CCRs. It was easier to identify family members who needed support at face-to-face consultations but not telephone consultations, especially if they had become informal carers:

*'So, like the lady that came with the gentleman ... I ended up giving her a depression questionnaire and I’m following her up ... as a person with her own needs, stepping outside of her husband’s palliative diagnosis*.' (Practice nurse, 009)

### Theme 3: changes to delivery of CCRs during the COVID-19 pandemic

The COVID-19 pandemic had a variable impact on CCR delivery. For some participants, CCR delivery did not occur as a result of reduced activity of oncology services in diagnosing and treating cancer cases. Other primary care staff continued with CCR implementation despite suspension of financial incentives:

*'When QoF was suspended during the pandemic actually the cancer care reviews were one of those things that we still carried on doing because we felt that that was important for patient care*.' (Salaried GP, 015)

However, sometimes CCRs were not prioritised before the COVID-19 pandemic because of other demands on primary care. Some thought that CCRs did not occur because patients were reluctant to attend the practice:

*'I would say our practice has changed in the way that we did provide the care. Because of COVID, patients themselves are scared, they are frightened to book an appointment ... I think that has caused the problem … there might be a bit of a gap*.' (Salaried GP, 006)

Despite this, more CCRs are being conducted at face-to-face appointments but patients retain autonomy on the type of consultation:

*'That can be up to them whether they want to come in or do it over the phone. People are coming back in, but with COVID they were generally over the phone but people are tending to come back in more.*' (Practice nurse, 010)

### Theme 4: ways to complement CCRs

Some clinicians described new ways to deliver CCR consultations other than the one clinician-to-one patient consultation model. This included group consultations which allow CCRs to be delivered with secondary care colleagues such as cancer nurse specialists and hospital consultants. There were added benefits of informal peer support and creating professional relationships between primary and secondary care:

' ... *we did a joint cancer care review group consultation with me and, one of the CNSs* [clinical nurse specialists – oncology] *and consultant colleagues ... It was very busy … but it was really good. And when there’s more scope for integrated working possibly*.' (Practice nurse, 010)

Another primary care staff member noted how a community cancer team consisting of a nurse and cancer support worker aimed to provide holistic cancer care. This team would accept referrals from patients as well as any other healthcare staff or service:

*'And then the referral is triaged there by the team, and then it is signposted to the best person in that team ... so if John has got a dog and he needs to go in and have chemotherapy, but has got nobody to sit with the dog, so he’s not going to have chemotherapy ... because that’s a very important part of his life. Then that cancer support worker will sort out that dogsitter. For the clinical side of it, then it would be the nurse, the clinical lead, that would then take that on*.' (Practice nurse, 009)

## Discussion

### Summary

The way that cancer was perceived by primary care staff — as a long-term condition and a multitude of different conditions simultaneously — made delivering CCRs challenging. In addition, primary care staff felt patients from diverse ethnic backgrounds had different views about cancer, which affected cancer care delivery. During the COVID-19 pandemic, there was a variable impact on CCR delivery because of the temporary suspension of cancer services and financial incentives for CCRs. The choice of CCR appointment modality via face-to-face or telephone was determined by patient choice and staff availability. Despite this, primary care staff noted the importance of face-to-face consultations for communication and visual cues. Before the CCR appointment, staff identified patient concerns by sending out instruments such as questionnaires. Staff took time to prepare before undertaking CCRs to best decide how to use appointment time. During appointments, primary care staff navigated several duties to provide holistic care including lifestyle advice, discussing sexuality, and explaining oncology letters. One specific role was filling in gaps in care which may be the result of unmet needs or healthcare system malfunction, termed *‘picking up the pieces’.* Templates guaranteed a care standard but were inflexible to individual patients’ needs. While CCRs are financially reimbursed they were a part of how cancer care was delivered annually. The impact of CCRs for patients was thought to be positive by providing an opportunity for care to occur as well as ‘acknowledgement’ of the difficulty of cancer diagnosis and/or treatment. However, not all patients had CCRs. Despite this, there is variation in cancer types, patient preferences, and associated needs. Family impact of CCRs was hard to identify especially during telephone consultations but may have been easier during in-person consultations. Group consultations and community cancer teams involving multi-sector working could help provide cancer care, including CCRs, in alternative ways. However, this would need commissioning and funding to ensure service sustainability.

### Strengths and limitations

This study captures the views of primary care staff delivering CCRs since changes in workforce, patient demand since 2015, and the COVID-19 pandemic.^11–13^ Purposive sampling ensured diversity in sex, staff seniority, and ethnicity. Only 20% of the interviewed sample were practice nurses, who may carry out more CCRs than GPs; the latter made up most of the sample. Other professionals who carry out CCRs such as nurse practitioners were not interviewed which may have influenced the reported experiences. Due to selection bias, there is a risk that this study preferentially presents findings from practitioners who had enough resources, such as time, and a special interest in cancer care.

Online interviews gave participants a choice of interview location, and it is unlikely that online interviews would be exclusionary in a digitally literate sample of primary care staff. Within an online setting itself, it may be more difficult to detect emotions such as distress or changes in body language compared to face-to-face interviews.^26^

While data collection was conducted by a novice researcher (DPG), there was supervision of coding quality, reflexivity, and ensuring quotes were representative of themes. Appropriate methodological guidance was followed throughout this study.^25^ Self-reporting of GP practice characteristics may have not been accurate, but independent verification for better accuracy was not possible because of limited resources.

### Comparison with existing research

Primary care staff perceptions about cancer as a chronic condition and the delivery of holistic care did reflect the literature concerning perceptions about people living with and beyond cancer in primary care.^27–29^ Acute needs, such as anxiety, were noted during interviews, but late complications of cancer and/or treatment were not mentioned, such as peripheral neuropathy or fear of cancer recurrence.^6^ This may be the result of a lack of focus on this aspect or lack of recognition of these aspects as complications of cancer treatment. Improvements to providing cancer care may involve changes in the provision of primary care and secondary care cancer follow-up services, such as dedicated cancer survivorship clinics or a multidisciplinary support team between primary and secondary care, which was suggested in this study.^30^

While the perception of cancer can affect delivery of cancer care, this study found that primary care staff delivered holistic cancer care. A systematic review^31^ of the role of the GP in long-term cancer care highlighted similar duties to those identified in this study, such as decoding jargon, psychosocial care, and partner support. Additional professional experience provided by practice nurses and a physician associate may have contributed to advice about sexuality, care coordination, and signposting to appropriate services such as social prescribers which are supplementary to those included in the review.

Of note, participants within this study felt that cancer was seen differently by people belonging to ethnic minority communities. A meta-synthesis looking at views of different ethnic minority communities globally found perceptions of cancer varied widely.^32^ Those from South Asian communities associated cancer with stigma and attributed cancer to ‘God’s will’ and to superstitious causes. A non-Eurocentric view on cancer can affect beliefs about the long-term effects of cancer and cancer treatment, health-seeking behaviours, and engagement with ‘conventional’ healthcare providers.

To ensure primary care staff provide cancer care, financial incentives were thought to be important to guarantee care provision before this study. However, this study found that such incentives did ensure CCR implementation, but CCRs were a small part of the cancer care delivered in primary care. There is limited evidence around the financial incentives (QoF) when applied to CCRs but there is some data on other clinical indicators. After the implementation of QoF, achievement rates of clinical indicators such as optimised blood pressure in hypertension rose, but achievement rates slowed down with time.^33,34^ This contrasts with non-incentivised indicators where there was no change. In contrast, removal of financial incentives is associated with decreased achievement rates for clinical indicators, as corroborated with fewer clinical tests requested.^35^ A complementary meta-synthesis of the experience of healthcare providers with the QoF between 2004 and 2018,^36^ suggests that QoF resulted in a loss in clinician autonomy and a distraction away from individual patient needs.^36^ This is consistent with the findings of this study, but some clinicians were motivated by a deeper sense of vocation rather than financial incentives.

### Implications for research and clinical practice

Policymakers will be encouraged to see the adoption of the new format of 3-month and 12-month CCRs which replaced the single 6-month CCRs that took place before 2020.^7^ This provides two patient contacts soon after a cancer diagnosis, reinforcing primary care as an available future support, but there is no way to measure quality of CCR. Funding for cancer as a long-term condition may support CCR implementation and associated support. Furthermore, the use of social prescribing as well as text messaging technology such as ‘Accurx’ indicates quick adoption of top-down policy changes in British primary care since the start of the COVID-19 pandemic.

Undergraduate and postgraduate educators should focus on how cancer can be seen as a long-term condition. This might include a holistic understanding of short-term and long-term effects on the individuals and their support networks. Another key area would be how people from diverse ethnic backgrounds may understand cancer, as this may differ from 'conventional', Eurocentric views.^32^

While this study highlighted the experience of primary care staff, further research^10^ could investigate the patient experience of CCRs which could help identify ways to improve CCRs. In general, the lack of research on CCRs highlights a need for the evaluation of CCRs in the healthcare service, and publication of associated results. Since CCRs do not provide any formal support to carers or caregivers, qualitative research may identify ways to provide support using both telephone and face-to-face consultations. Any new interventions to provide patient or caregiver support would require dialogue with primary care staff and policymakers before implementation.

## Data

The dataset relied on in this article is available from the corresponding author on reasonable request.
